# Analysis of cluster randomised stepped wedge trials with repeated cross-sectional samples

**DOI:** 10.1186/s13063-017-1833-7

**Published:** 2017-03-04

**Authors:** Karla Hemming, Monica Taljaard, Andrew Forbes

**Affiliations:** 1 Institute of Applied Health Research, University of Birmingham, Birmingham, B15 2TT UK; 2 Clinical Epidemiology Program, Ottawa Hospital Research Institute, 1053 Carling Avenue, Ottawa, ON K1Y4E9 Canada; 3 Department of Epidemiology and Community Medicine, University of Ottawa, Ottawa, ON Canada; 4 School of Public Health and Preventive Medicine, Monash University, Melbourne, VIC Australia

**Keywords:** Stepped wedge, Cluster randomised trial, Analysis, Secular trends

## Abstract

**Background:**

The stepped wedge cluster randomised trial (SW-CRT) is increasingly being used to evaluate policy or service delivery interventions. However, there is a dearth of trials literature addressing analytical approaches to the SW-CRT. Perhaps as a result, a significant number of published trials have major methodological shortcomings, including failure to adjust for secular trends at the analysis stage. Furthermore, the commonly used analytical framework proposed by Hussey and Hughes makes several assumptions.

**Methods:**

We highlight the assumptions implicit in the basic SW-CRT analytical model proposed by Hussey and Hughes. We consider how simple modifications of the basic model, using both random and fixed effects, can be used to accommodate deviations from the underlying assumptions. We consider the implications of these modifications for the intracluster correlation coefficients. In a case study, the importance of adjusting for the secular trend is illustrated.

**Results:**

The basic SW-CRT model includes a fixed effect for time, implying a common underlying secular trend across steps and clusters. It also includes a single term for treatment, implying a constant shift in this trend under the treatment. When these assumptions are not realistic, simple modifications can be implemented to allow the secular trend to vary across clusters and the treatment effect to vary across clusters or time. In our case study, the naïve treatment effect estimate (adjusted for clustering but unadjusted for time) suggests a beneficial effect. However, after adjusting for the underlying secular trend, we demonstrate a reversal of the treatment effect.

**Conclusion:**

Due to the inherent confounding of the treatment effect with time, analysis of a SW-CRT should always account for secular trends or risk-biased estimates of the treatment effect. Furthermore, the basic model proposed by Hussey and Hughes makes a number of important assumptions. Consideration needs to be given to the appropriate model choice at the analysis stage. We provide a Stata code to implement the proposed analyses in the illustrative case study.

**Electronic supplementary material:**

The online version of this article (doi:10.1186/s13063-017-1833-7) contains supplementary material, which is available to authorized users.

## Background

Stepped wedge cluster randomised trials (SW-CRTs), that is, trials in which clusters are randomised to become exposed to an intervention sequentially over time [1–3], are rapidly increasing in popularity. In the conventional version of this design, which is the focus in this paper, all clusters are initially observed in the unexposed condition; then at usually regular intervals, one or more clusters are sequentially randomised to become exposed to the intervention and remain exposed for the duration of the study. The study continues until all clusters are exposed. Outcomes may be assessed on cross-sectional samples of individuals from each cluster at multiple discrete time points, on the same cohort followed over time, or as mixture of the two. In this paper we focus on the cross-sectional design. Examples of interventions evaluated using the stepped wedge design include changes to the way that health services are delivered and health care professional training interventions [4–7].

A characteristic feature of this design is that the evaluation happens over an extended period of time in which the proportion of clusters exposed to the intervention gradually increases. This means that the control clusters will, on average, contribute observations from an earlier calendar time than the intervention clusters. In evaluations of policy changes and service delivery interventions, there may be secular changes in the outcome caused by external forces such as changes in the way that care is delivered. Thus, calendar time may be associated with the outcome in addition to its association with exposure to the intervention and so is a potential confounder [3, 5]. Stepped wedge studies that do not adjust for time (i.e. do not allow for the possibility of secular trends) are, therefore, potentially biased [3]. Whilst a recent, small, systematic review identified that 8 of a total of 10 studies adjusted for secular trends [8], results from our own larger review of 32 published trials identified that only 17 (53%) clearly allowed for the secular trends within the primary estimate of the treatment effect (unpublished result) [9]; and another review has shown that only 61 out of 102 trials mentioned time effects in the analysis [10]. In the early history of cluster randomised trials it was not unusual to see trial results published without allowing for clustering, thus yielding results which were overly precise. This remained a prevalent problem for years [11–13]. A similar situation has already started to arise in the SW-CRT literature wherein trialists are failing to adjust for time [14]. In fact, failure to account for time has even appeared in the recent methodological literature [15]. This is potentially a greater problem than failing to adjust for clustering, as it has implications for bias as well as for precision.

Another important consideration in stepped wedge trials is that the basic analytical model proposed by Hussey and Hughes [16] makes a number of assumptions. These assumptions may be under-appreciated by some clinical trialists, as sensitivity to their departure is rarely investigated or considered at the analysis stage. Yet, these model assumptions are different and more restrictive than assumptions made in the related framework proposed for the analysis of cluster cross-over trials [17]. Furthermore, whilst others have started to appreciate these assumptions when determining the sample size needed in a stepped wedge trial [18], there is no single paper which addresses all of these issues with respect to statistical analysis.

In this paper, our objectives are to (1) review the assumptions implicit in the basic Hussey and Hughes model and to consider their implications; (2) consider how these models can be extended to allow for deviations from assumptions, including heterogeneity in the secular trend and in the treatment effect and (3) discuss the importance of adjusting for secular trends. We present a case study to demonstrate the application of the proposed model extensions and to show how trial conclusions can differ as a result of adjusting for secular trends.

## Methods

**Fig. 1:**
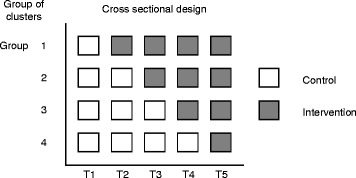
Schematic illustration of the stepped wedge cluster randomised trial

The defining feature of the SW-CRT is that clusters are randomised to initiate the intervention at different points in time. We define the points at which clusters are randomised to cross from the control to intervention as ‘steps’. Figure 1 presents a schematic illustration of the SW-CRT. The simplest stepped wedge study design has one cluster crossing over at each step (i.e. has the same number of steps as clusters) and has one additional measurement point to the number of steps (i.e. a preintervention measurement). Where more than one cluster switches at each step we refer to this as a group of clusters. Variations on the common design include multiple measurement points before any randomisation occurs or after all clusters have switched, steps between which no measurements are taken, and steps between which multiple measurements are taken.

### Case study: a SW-CRT to evaluate a training package to promote sweeping of the membranes

**Fig. 2:**
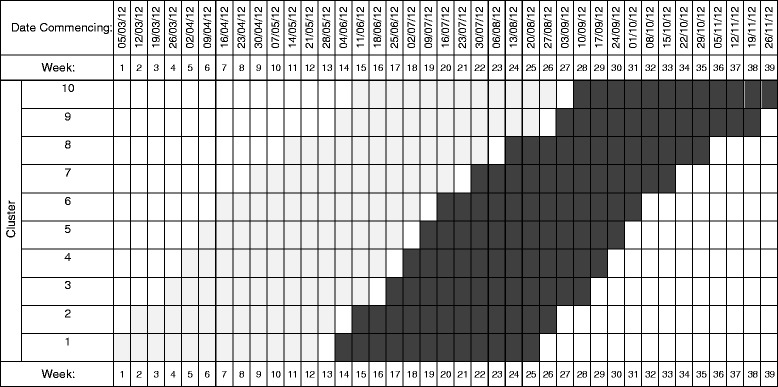
Schematic representation of study design for case study

The UK body, the National Institute of Health and Clinical Excellence (NICE), recommends sweeping of the membranes at term to reduce induction of labour, but it is known that many women do not receive a membrane sweep. A pragmatic SW-CRT of 10 midwifery teams (with teams forming the clusters) was undertaken to evaluate whether a training package to promote sweeping of the membranes in post-term women is effective [19]. These 10 midwifery teams were split across two different hospitals, with 5 teams serving each hospital and hospital used as a stratification factor in the randomisation. The timing of the introduction of the intervention in each cluster was randomly allocated and evaluated using a slightly modified version of the stepped wedge cluster design (Fig. 2). Time was categorised into 39 time periods (each representing a week) with each birth occurring over the study duration being classified into the appropriate cluster by time period. The study included 2864 low-risk women over 39 weeks’ gestation and ran from 5 March 2012 to 26 November 2012. The primary outcome was whether or not the women had a membrane sweep. The next section commences with analysis methods for continuous outcomes then describes natural extensions for binary outcomes – applicable to our case study.

### Basic model

Hussey and Hughes [16] have suggested a model-based approach for analysing data from a cross-sectional SW-CRT. This approach was proposed for continuous outcome variables and has been commonly used at the design stage of these studies [9]. It involves fitting a linear mixed model:

$$ {Y}_{i j l}\kern0.5em =\kern0.5em {\beta}_0\kern0.5em +\kern0.5em {\beta}_j\kern0.5em +\kern0.5em \theta {X}_{i j}\kern0.5em +\kern0.5em {u}_i\kern0.5em +\kern0.5em {e}_{i j l,} $$

where *i* indexes the cluster, *j* indexes time and *l* indexes the individual, with *Y*_*ijl*_ a continuous outcome. The term *u*_*i*_ ~ *N*(0, *σ*_*u*_^2^) represents a cluster-level random effect, *e*_*ijl*_ ~ *N*(0, *σ*_(*e*)_^2^) the individual error terms, *X* is a binary variable representing exposure to the treatment (1 for treatment, 0 otherwise), *θ* is the treatment effect. Following Hussey and Hughes, *β*_*j*_ represents a fixed categorical effect to model the underlying secular trend (*β*_*T*_ = 0 for identifiability where *T* is the total number of measurement periods) and *β*_0_ an intercept term which represents the population average during the first time interval. It is convenient to think of *u*_*i*_ representing the time invariant deviation of the ith cluster from the population average.

The intracluster correlation coefficient (ICC) which, under this model, is assumed constant over time, is then:

$$ \rho \kern0.5em =\kern0.5em \frac{\sigma_u^2}{\sigma_u^2\kern0.5em +\kern0.5em {\sigma}_e^2}. $$

Hussey and Hughes consider models for continuous outcomes with an identity link (to report mean difference). But, this approach naturally extends to *generalised* linear mixed models for other outcome types (e.g. binary as in the case study), other link functions (e.g. logit) and consequently, other scales for the treatment effect (e.g. odds ratio (OR)).

It is important to recognise the underlying assumptions of the basic Hussey and Hughes model, both with respect to fixed and random components. Most notably, the model includes a fixed effect for time (implying a common underlying secular trend across all clusters) and a single term for treatment (implying a constant shift in this trend under the treatment). In the basic model the effect of time is modelled as a categorical variable which allows for a piecewise secular trend. The model additionally implies a simple correlation structure, whereby the correlation between any two observations in the same cluster is the same regardless of treatments administered and duration between the time periods of the observations.

The model also assumes that the data obtained, at multiple discrete time points, are on different individuals. However, whilst the data are assumed measured at discrete points in time in this model, the actual measurements might be obtained in continuous time (as in our case study) but categorised into time intervals defined by the steps.

We first consider extensions in which the assumption about the secular trend is relaxed and then move on to consider extensions in which the treatment effect is allowed to vary in some way. These extensions are incorporated either by extending the fixed or random components of the model. Each extension makes implicit modifications to the assumptions about the correlation structures – we outline these along the way. For clarity we build the models up in a stepwise process. For notational clarity, fixed effects are represented using Greek letters and random effects using lower case Roman letters. We use *θ* throughout to denote the treatment effect, sometimes with a subscript to indicate a treatment effect that varies across some factor.

### Extensions to allow different secular trends

In the basic Hussey and Hughes model, a homogeneous secular trend is assumed across all clusters. Whilst the underlying secular trend may not be of substantive interest, a misspecified secular trend may lead to biased treatment effects or biased standard errors. Random deviations (across clusters) from the underlying secular trend have already been proposed in the context of cluster cross-over designs and cluster trials with repeated cross-sectional measurements [17, 20, 21]. Such deviations have also been proposed in stepped wedge trials in the context of design and efficiency considerations [22, 23]. We consider extensions to the basic model to allow for variation in the secular trend across clusters, first using fixed effects and then using random effects.

#### Model extension A: varying secular trends across strata of clusters – using fixed effects

Secular trends might vary across clusters or defined subgroups of clusters (i.e. strata). For example, in our case study involving stratified allocation of midwifery teams within two separate hospitals, it may be reasonable to allow for different secular trends in each hospital. This can be accomplished by adding a fixed-effect interaction between time and stratum to the basic model:

$$ {Y}_{i(s) j l}\kern0.5em =\kern0.5em {\beta}_0\kern0.5em +\kern0.5em {\beta}_j\kern0.5em +\kern0.5em \theta {X}_{i(s) j}\kern0.5em +\kern0.5em {\gamma}_{j s}{Z}_{i(s)}\kern0.5em +\kern0.5em {u}_i\kern0.5em +\kern0.5em {e}_{i(s) j l}, $$

where *Y*_*i*(*s*)*jl*_ indicates that cluster *i* is nested within stratum *s*, *Z*_*i*(*s*)_ is an indicator for stratum and γ_*js*_ represents the fixed time by stratum interaction (constrained such that γ_*js*_ is 0 for the last strata). One natural choice might be to treat any stratification grouping used in the randomisation procedure as strata. This model extension, modifying the fixed-effects components only, makes no modifications to the underlying correlation structure.

#### Model extension B: varying secular trends across clusters – using random effects

Secular trends might also be allowed to vary randomly across clusters, rather than across fixed subgroups of clusters. This can be incorporated by extending the random-effects components to allow a random interaction between time and cluster *v*_*ij*_:

$$ {Y}_{i j l}\kern0.5em =\kern0.5em {\beta}_0\kern0.5em +\kern0.5em {\beta}_j\kern0.5em +\kern0.5em \theta {X}_{i j}\kern0.5em +{u}_i\kern0.5em +\kern0.5em {v}_{i j}\kern0.5em +\kern0.5em {e}_{i j l}, $$

where *v*_*ij*_ ~ *N*(0, *σ*_*v*_^2^) and where *v*_*ij*_ is assumed independent to *u*_*i*_. In this model, each cluster has a different random effect at each time point and thus a different deviation from the average secular trend – although the degree of variation (as indicated by *σ*_*v*_^2^) is sampled from the same distribution at all time points and for all clusters [24]. It is convenient to think of *v*_*ij*_ representing the variation within a cluster due to time varying characteristics of the cluster.

One implication of including a random time by cluster interaction is that it allows the intracluster correlation to depend on whether observations are in the same time period or in different time periods. In particular, it allows for two different correlation coefficients:

$$ {\rho}_W\kern0.5em =\kern0.5em \frac{\sigma_u^2\kern0.5em +\kern0.5em {\sigma}_v^2}{\sigma_u^2\kern0.5em +\kern0.5em {\sigma}_v^2\kern0.5em +\kern0.5em {\sigma}_e^2} $$

and

$$ {\rho}_I\kern0.5em =\kern0.5em \frac{\sigma_u^2}{\sigma_u^2\kern0.5em +\kern0.5em {\sigma}_v^2\kern0.5em +\kern0.5em {\sigma}_e^2}, $$

where *ρ*_*W*_ represents the within-cluster, within-period correlation and *ρ*_*I*_ the interperiod (same cluster) correlation, with the restriction that *ρ*_*I*_ ≤ *ρ*_*W*_. It is intuitive that there may be a stronger correlation between observations within the same cluster and period than between observations within the same cluster but in different periods. We note that this model specification is analogous to specifications proposed for two period cluster randomised cross-over trials [17]. An alternate but equivalent specification is with the use of *ρ*_*I*_/*ρ*_*W*_ rather than *ρ*_*I*_, which has been termed the cluster autocorrelation [25]. On the downside this model assumes that the correlation between two observations taken at different times is the same irrespective of how far apart in time those observations are made.

### Extensions to allow for treatment-effect heterogeneity across clusters

In multicentre individually randomised trials, in which centres can have both treatment and control observations, treatment-effect heterogeneity can be investigated by including a treatment by centre interaction [26]. In stepped wedge trials, because each cluster is both exposed and unexposed, it is also possible to model treatment by cluster interactions. Others have already considered the issue of allowing for treatment by cluster heterogeneity in power calculations for stepped wedge trials [18, 27], although here we propose slightly different model parameterisations which make less restrictive assumptions. Extensions to allow for treatment-effect heterogeneity are considered in model extensions C to E below, first using fixed effects and then using random effects.

#### Model extension C: varying treatment effect across strata of clusters – using fixed effects

In addition to examining heterogeneity in secular trends across strata, it might also be reasonable to examine treatment-effect heterogeneity across strata. So, in our case study set across two different hospitals, there may be pragmatic interest in how the treatment effect varies across these hospitals. This can be incorporated by including a fixed-effect interaction between treatment and strata, to model the different treatment effects in each stratum/strata:

$$ {Y}_{i(s) j l}\kern0.5em =\kern0.5em {\beta}_0\kern0.5em +\kern0.5em {\beta}_j\kern0.5em +\kern0.5em \theta {X}_{i(s) j}\kern0.5em +\kern0.5em {\theta}_s{X}_{i(s) j}{Z}_{i(s)}\kern0.5em +\kern0.5em {u}_i\kern0.5em +\kern0.5em {e}_{i(s) j l}, $$

using the same notation as earlier, where *Z*_*i*(*s*)_ represents a stratum covariate and now *θ*_*s*_ represents a fixed-effect interaction between treatment status and stratum (again constrained such that *θ*_*s*_ is 0 for the last strata).

The model includes a single underlying secular trend for all strata, but a different shift in treatment effect (constant over time) for each stratum. Interest in treatment-effect variation by strata may have very practical implications, as it allows addressing the question of whether or not a treatment was effective in a subgroup of clusters.

#### Model extension D: treatment-effect heterogeneity across clusters – using random effects

Where interest lies in examining treatment-effect heterogeneity across clusters themselves, rather than fixed subgroups of clusters, this can be achieved by including a random interaction between treatment and cluster. We parameterise this model as follows:

$$ {Y}_{ij l}\kern0.5em =\kern0.5em {\beta}_0\kern0.5em +\kern0.5em {\beta}_j\kern0.5em +\kern0.5em \theta {X}_{ij}\kern0.5em +\kern0.5em {u}_{Ti}{X}_{ij}\kern0.5em +\kern0.5em {u}_{Ci}\left(1-{X}_{ij}\right)\kern0.5em +\kern0.5em {e}_{ij l}. $$

The two random-effect terms *u*_*Ti*_ and *u*_*Ci*_ represent the random interaction between treatment status and cluster. These two random effects have a different variance parameter for those exposed (*u*_*Ti*_ ~ *N*(0, *σ*_*T*_^2^)) and those unexposed (*u*_*Ci*_ ~ *N*(0, *σ*_*C*_^2^)); and importantly, a covariance between these two random effects (*σ*_*TC*_). Note that this model allows the variability between clusters to differ between the intervention and control periods.

This parameterisation implies different pairwise correlations within clusters, depending on whether observations are both unexposed, both exposed, or one exposed and one unexposed. In particular, it implies:

$$ {\rho}_{C T}\kern0.5em =\kern0.5em \frac{\sigma_{C T}}{\sqrt{\left({\sigma}_C^2\kern0.5em +\kern0.5em {\sigma}_e^2\right)\left({\sigma}_T^2\kern0.5em +\kern0.5em {\sigma}_e^2\right)}},\kern0.5em {\rho}_{C C}\kern0.5em =\kern0.5em \frac{\sigma_C^2}{\sigma_C^2\kern0.5em +\kern0.5em {\sigma}_e^2},\kern0.5em \mathrm{and}\kern0.5em {\rho}_{T T}\kern0.5em =\kern0.5em \frac{\sigma_T^2}{\sigma_T^2\kern0.5em +\kern0.5em {\sigma}_e^2}, $$

where *ρ*_*CT*_ represents the correlation between two observations in the same cluster but with different exposures; and *ρ*_*TT*_ and *ρ*_*CC*_ the correlations between two observations in the same cluster and same treatment exposures. There is no restriction as to which of these correlations are larger due to the inclusion of the covariance term.

### Extensions to allow for treatment-effect heterogeneity across time

In addition to treatment heterogeneity across clusters, treatment effects might vary with time. Because the SW-CRT is run over multiple time periods it is possible to investigate treatment by time interactions. Others have considered delayed treatment effects (i.e. an interaction between treatment and time since the cluster was exposed) [27]. We consider extending the basic model for treatment heterogeneity that varies over step. That is, we consider a fixed-effect interaction between time period and treatment.

#### Model extension E: treatment-effect heterogeneity across time – using fixed effects

Interest might lie in determining whether treatment effects vary across time. This can be achieved by extending the basic Hussey and Hughes model to include a fixed interaction between treatment status and time period (step). We parameterise this model as follows:

$$ {Y}_{i j l}\kern0.5em =\kern0.5em {\beta}_0\kern0.5em +\kern0.5em {\beta}_j\kern0.5em +\kern0.5em \theta {X}_{i j}\kern0.5em +\kern0.5em {\theta}_j{X}_{i j}\kern0.5em +\kern0.5em {u}_i\kern0.5em +\kern0.5em {e}_{i j l}. $$

This model again assumes that each cluster follows the same piecewise secular trend, but this model allows the treatment effect to differ at each randomisation step in the study. Again for identifiability, the treatment effect in the last time period is constrained to be 0 (*θ*_*T*_ = 0).

Note that this model extension modifies the fixed-effects components only and makes no modifications to the underlying correlation structure. We note that this fixed-effect interaction will be estimable only at time periods in which there are both exposed and unexposed observations. So, in our case study, this will be across weeks 13 to 26.

## Results

Each of the models described above was fitted to the case study. Recall that the primary aim in the case study was to evaluate whether there is a difference in the probability of women’s membranes being swept during labour both before and after the intervention. We fitted several models: an inappropriate model that does not account for time; the Hussey and Hughes basic model; and each of the model extensions described in the previous section. We report ORs, and so fit the logistic regression model.

These models can be fitted in any statistical package, such as SAS, R, or Stata. We used Stata 14 to fit models using the melogit function using maximum likelihood methods. This function uses mean-variance adaptive Gauss-Hermite quadrature (Stata’s default estimation method). We used the default number of integration points (7) and default starting values. For one model (extension D) we observed convergence difficulty using the melogit function and used the xtmelogit function instead – which is recommended by Stata as an alternative in cases of variance components being near the boundary of the parameter space.

We estimate the ICCs on the logistic scale [28] using the estat function where possible (although in more complicated models we estimate the ICC simply by taking the ratio of variances and for these cases no confidence interval for the ICC is provided). We provide sample Stata code as Additional file 1. The results are summarised in Table 1. Of note there are 10 randomisation steps, but 13 time periods in which there are both control and intervention observations, for which a treatment by time interaction is potentially estimable.

**Table 1:** Estimates of treatment effect from the Hussey and Hughes model; and model extensions

Model	Unexposed to intervention	Exposed to intervention	Odds ratio (95% CI)	ICC (95% CI)
	*N* = 1420	*N* = 1367		
Number swept	629 (44.3%)	634 (46.4%)		
Unadjusted for time models				
Naïve model			1.11 (0.95, 1.30)	0.069 (0.028, 0.161)
Time-adjusted models				
Basic Hussey and Hughes model			0.78 (0.55, 1.12)	0.073 (0.030, 0.168)
Model extensions				
A: Time by strata interaction (FE)			0.80 (0.55, 1.17)	0.075 (0.030, 0.176)
B: Time by cluster interaction (RE)^a^			0.79 (0.55, 1.14)	0.073 (0.030, 0.168)
				0.078 (0.032, 0.177)
C: Treatment by strata interaction (FE)^b^			0.85 (0.58, 1.23)	0.066 (0.026, 0.156)
			0.80 (0.57, 1.13)	
D: Treatment by cluster interaction (RE)^c^			0.76 (0.52, 1.12)	0.016; 0.045; 0.027
E: Treatment by time interaction (FE)^d^			0.86 (0.21, 3.49)	0.075 (0.030, 0.171)

*CI* confidence interval, *ICC* intracluster correlation, *FE* fixed-effect interaction, *RE* random-effect interaction. ^a^ICCs presented are within same cluster same period; and same cluster different period; ^b^Treatment effects for two strata (hospital A and hospital B); ^c^ICCs presented are within same cluster both treated; and same cluster both untreated; and same cluster different treatment; ^d^Treatment effect given is at mid study week 20 – others are depicted in Fig. 4. Note the ICC is reported on the logistic scale and so is not to be used for planning purposes. All models adjust for clustering

Note the summaries of number and proportion swept in first two columns are unadjusted for time and so should not be interpreted as representative of the treatment effect. Estimates from model D are using xtmelogit as melogit failed to converge

### Basic model and implications of naïve (unadjusted for time) estimates

**Fig. 3:**
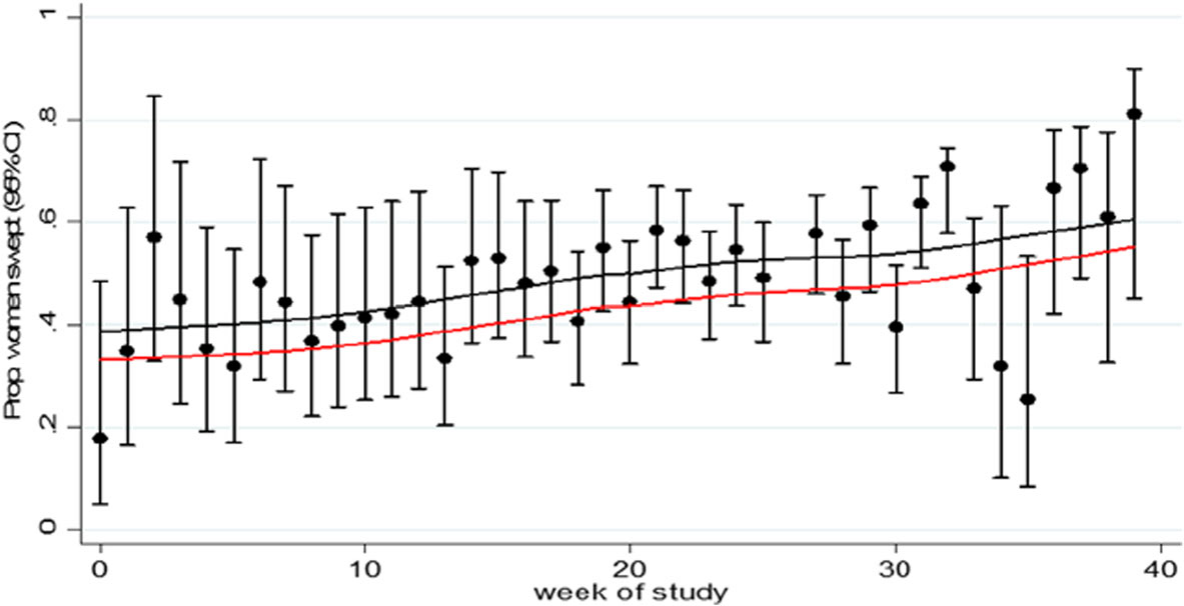
Model-based estimate of underlying temporal trend in primary outcome over duration of trial in unexposed clusters (*black line*) and model-based estimated of outcome in intervention periods (*red line*) – basic model for the case study. Point estimates and 95% CI for each step with smoothed (LOWESS) line overlaid (*black* control; *red* intervention)

Based on the observed data, there was a very small increase in the proportion of women swept in the period after exposure to the intervention (44.3% versus 46.4%). The naïve analysis, which accounts for treatment and a random effect for cluster (but not time), found a positive but nonstatistically significant effect of the intervention (OR 1.11; 95% CI 0.95 to 1.30). When modelling time as a categorical variable in those not exposed to the intervention there was some evidence of a small increasing trend over time in the proportion of women being swept (Fig. 3). Most notably, after adjusting for this secular trend, the estimated odds ratio shifted from favouring the intervention to favouring the control. In particular, the adjusted analysis found a negative, but nonstatistically significant effect of the intervention (OR 0.78; 95% CI 0.55 to 1.12). The estimated ICC was 0.073 (95% CI 0.030, 0.168).

### Models allowing for different secular trends

The case study was set across two different hospitals and so it is very possible that background trends differed between these two hospitals. We therefore allowed secular trends to vary across these two hospitals (model extension A). We observed no clear difference in underlying secular trends between the two hospitals (Figure not shown): inclusion of this term resulted in a nonsignificant interaction and the overall conclusions about the treatment effect did not change substantially (Table 1). We also fitted model extension B, allowing for random variation in the secular trend across the 10 clusters, as it seemed intuitive that as the study was conducted over 40 weeks that some time-dependent random variation was to be expected. Whilst we found that the correlation between observations in different time periods (0.073; 95% CI 0.030, 0.168) was slightly lower than between observations within the same time period (0.078; 95% CI 0.032, 0.177), the estimated treatment effect did not change substantially (Table 1). When fitting these model extensions, which allowed for differing secular trends, we observed an increase in the width of the confidence intervals, reflecting a decrease in precision associated with fitting a more flexible model.

### Models allowing for treatment-effect heterogeneity

**Fig. 4:**
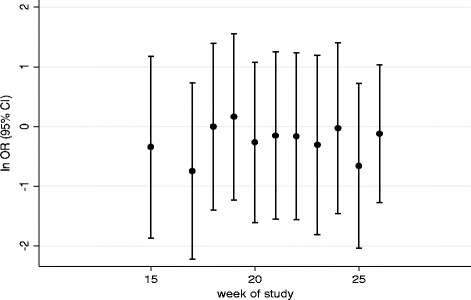
Model-based estimate of treatment effect (ln odds ratio, OR) over duration of trial. Point estimates and 95% CI for each time period in which observations were both exposed and unexposed to intervention

When we allowed for a fixed-effect interaction to investigate treatment-effect heterogeneity across the two hospitals, we found that the interaction was not significant and the treatment effects similar between the two strata (Table 1, model extension C). In the model in which a random treatment by cluster interaction was included (model extension D), we observed some indication that the correlation was lower between observations which were treated (point estimate 0.016) than between observations which were not treated (point estimate 0.045). However, the treatment estimate changed little on allowing for this extra heterogeneity (Table 1). Allowing for treatment by time (step) interactions (model extension E) was uninformative as confidence intervals were very wide (Fig. 4). Again, all confidence intervals widened reflecting the decrease in precision associated with fitting these more complex models.

## Discussion

The SW-CRT is a novel study design which is increasing in popularity and can be potentially valuable in the evaluation of service delivery and policy interventions [1, 2]. A recent systematic review found that a substantial proportion of stepped wedge studies published to date had failed to adjust for secular trends in the estimated treatment effect [9, 10]; and recent papers in high-impact journals have also failed to adjust for this confounder [14, 29]. We have demonstrated in this paper the consequences of not adjusting for time. Underlying secular trends in those clusters unexposed to the intervention in our example illustrates the real possibility of changes over time in the outcomes, irrespective of any intervention. Furthermore, the common modelling framework proposed for the analysis of the SW-CRT by Hussey and Hughes – which does allow for secular trends – makes a number of assumptions. These include the assumption of a common underlying secular trend and a simple shift under treatment exposure across all clusters and all time periods. In this paper, we demonstrated how to explore sensitivities to deviations from these basic model assumptions. Whilst our case study did not prove to be sensitive to these model assumptions, it is important to note that this is unlikely to be the case in all studies.

### The impact of secular trends

In our example, we observed how the primary outcome increased over time in clusters unexposed to the intervention. The implication of this was that an intervention which prima facie appeared effective, albeit with a small impact, actually transpired to be suggestive of an intervention which had an effect in the opposite direction. There are several possible explanations for this finding [5]. Firstly, the fully adjusted confidence intervals did not rule out that the intervention may indeed be beneficial (i.e. the result was not statistically significant). Alternatively, contamination might be an explanation; that is, the unexposed clusters becoming exposed to the intervention before their randomised cross-over point. But, it might be the case that external to this intervention and study, other nationwide initiatives began, sometimes described as a rising tide [30].

### Modelling secular trends

Because of the inherent imbalance in treatment over time it is imperative that the primary analysis of a SW-CRT adjusts for time irrespective of whether it is statistically significant. Time is a strong candidate for having a confounding effect in almost every SW-CRT and this logical reasoning should dictate its criteria for inclusion rather than a reliance on statistical significance testing, or lack of, which is not useful for determination of confounding [31]. In our example we observed the effect of calendar time to be reasonably linear. Modelling a linear trend will require fewer degrees of freedom than modelling as a categorical variable and so should provide increased precision for the treatment effect. With a large number of steps, or where time is recorded continuously, it should also be possible to model the secular trend using quadratic or cubic terms, or even fractional polynomials [32] or restricted cubic splines. With more than about four or five measurement occasions (or when time is measured continuously), fitting a parametric trend for time might result in more powerful statistical tests assuming that the modelled parametric trend actually provides a reasonable approximation to the true underlying shape [33].

### Extensions from the basic Hussey and Hughes model

The Hussey and Hughes model is becoming standard for use in the analysis of the SW-CRT. In our review we have identified that of the 32 published trials – 17 of these adjusted for secular trends and of these 7 used the Hussey and Hughes model [9]. We did not identify any studies that considered deviations from the basic model – either by modelling more flexible secular trends or exploring treatment heterogeneity. Yet, the Hussey and Hughes model makes some important implicit assumptions, namely, that the underlying secular trend is identical across all clusters, and that the treatment produces a constant shift in this trend which is identical across all clusters and time points. It is important that these assumptions are recognised so that their appropriateness can be considered. We have outlined these assumptions and considered how they may be extended. We considered extensions in which the secular trend was allowed to vary across clusters and the treatment effect was allowed to vary across clusters and time [34].

Correctly modelling the secular trend is important, otherwise the secular change might be mistakenly attributed to the intervention. Modelling treatment-effect heterogeneity might be considered less important if interest is in the average treatment effect but will affect the precision of the treatment effect. Furthermore, implicit in a model that assumes a single random intercept for the cluster is that the correlation between two observations in the same cluster is independent of their treatment status, an assumption that seems untenable. We observed a slight decrease in precision when we fitted these more complex models and this is to be expected more generally and is a reflection of model complexity. This will of course have implications on power – to the extent that appropriate consideration of these model extensions at the power stage will result in an increase in sample size needed [22, 23].

We considered both extensions using random- and fixed-effect parameters. For the fixed-effects models we group clusters into strata. In our case study, the stratum is hospital as the study was set across two hospitals. Other choices for strata will be dependent on context. Fixed effects might be appropriate where there is a clear clinical interest in the effect of these strata. So, in our case study a clinical interest in whether the treatment effect varies by these particular hospitals. There are clearly downsides though to the use of fixed effects as it will quickly reduce the degrees of freedom when the number of strata becomes large. Furthermore, when modelling the secular trend, the secular trend might be expected to vary across all clusters and not just across strata.

In our case study we did not find model choice to have an impact on the treatment effect or its precision (other than the model in which time was not included). This cannot and should not be taken as a generalisable result – we expect model misspecification to have a large impact in some situations. Indeed, model misspecification in generalised linear mixed models is known to be important, as others have demonstrated in clustered data [35]. Misspecification can arise because of either misspecification of the model for the mean or misspecification of the correlation structure. Misspecification of the mean might arise because of cluster-specific variation in secular trends perhaps, but these sorts of misspecifications depend on the study and so are difficult to investigate generically. Misspecification of the correlation structure, however, can be investigated generically – and so, for example, future simulation studies should investigate the impact on bias and precision of treatment effects estimated when there is decay in the correlation overtime but where this is ignored at the analysis stage.

### Limitations

Although we have described extensions to the Hussey and Hughes approach, we did not examine the consequences of model misspecification on bias, coverage and power using simulations. There were also other extensions to the basic model which we did not consider. For example, an extension that we did not consider was to impose a structure on the cluster-level covariance over time, such as linear or autoregressive decay in correlations over time. However, we did allow for an interaction between the time trend and some strata variable, but this will only be feasible where there is a natural choice for strata as it is not possible to model an interaction between cluster and time as a fixed effect [36].

We limited our consideration to the generalised linear mixed model, but generalised estimating equations (GEEs) also allow for nonindependence in cluster trials [37]. Within the context of the SW-CRT, finite sample GEE correction methods have been proposed, but these are not widely available in Stata yet (though they are available in R) [38]. Including cluster as a fixed effect is a possibility; as too is an analysis using cluster-level means [39]; or something described by others as a vertical analysis [40]. Vertical analyses involve separate analyses at each time period and pooling time-specific treatment effects – using some sort of weighting methods. Vertical analyses are likely to make fewer assumptions, but at a cost of being less powerful [8].

We have also identified some more technical issues along the way. We observed model convergence issues with one of the model extensions (model D), which we were able to overcome using a different method of optimisation. We were also unable to obtain treatment by time interactions for all of our randomisation step points – due to scarcity of data. These complex models were fitted to data with only 10 clusters, and without any small sample correction factors. Others have shown that this may have implications on bias and coverage, especially when using GEEs [38]. Finally, we reported correlations on the logistic scale. It is well known that for planning parallel cluster trials the ICC needed is on the proportions scale. However, whether the same principals apply to other correlations (such as interperiod correlations) is unknown. We also did not report confidence intervals for all correlation parameters, due to current statistical package limitations.

We have stopped short of providing recommendations for which of these models should be used for the primary analysis, have not considered issues of multiple testing nor consequences of lack of a priori model prespecification. Whilst these issues are very important, our aim has been to raise awareness of the assumptions implicit in the Hussey and Hughes model and provide some but not all possible model extensions. Future work lies in determining consequences of model misspecification, which can then help with recommendations for primary analysis. We would recommend that the model used should be selected prior to analysing the data and should not be data driven. Until the consequences of model misspecfication are known, we would recommend that the primary analysis consist of the Hussey and Hughes model, and sensitivity of results to departures from the model (as outlined here) should be considered and reported.

## Conclusions

The SW-CRT is particularly appealing as it offers a means of conducting a randomised trial within a naturalistic setting. In order to successfully determine the effectiveness of an intervention evaluated using a SW-CRT, it is necessary to use appropriate analysis techniques because simple summary statistics (grouped by those exposed and not exposed to the intervention) will not be a fair summary of the effect of the intervention. In essence, this means that there is a necessity for allowing for underlying secular trends and it is this time-adjusted effect of the intervention which is the unbiased estimate of how the intervention works. This adjusted effect, as shown in our examples, can be quite different to the unadjusted effect.

Furthermore, whilst the model proposed by Hussey and Hughes is becoming increasingly used at the analysis stage, which does indeed adjust for time, it is important to appreciate the assumptions implicit within this model – both the assumptions that are being made about secular trends and treatment effects; and the implied assumptions on the correlation structure. Further work, likely using simulation studies, is needed to determine if model misspecification has important consequences in terms of bias or standard error of the intervention effect estimate.

## Additional file

Additional file 1: Stata code to implement proposed models. (DOCX 14 kb)

## Abbreviations

CI: Confidence interval
CRT: Cluster randomised trial
ICC: Intracluster correlation
RCT: Randomised controlled trial
SW-CRT: Stepped wedge cluster randomised trial
