# Supplementary material for: Analysis of cluster randomised stepped wedge trials with repeated cross-sectional samples
Source: Trials. 2017 Mar 4;18:101. doi: 10.1186/s13063-017-1833-7 (PMC5336660; doi:10.1186/s13063-017-1833-7)
Supplement: Additional file 1: — Stata code to implement proposed models. (DOCX 14 kb) [file 13063_2017_1833_MOESM1_ESM.docx]

**Additional file 1: Stata code to implement proposed models**

***Variable definitions**

"Treat" represents the treatment indicator (binary)

"Cluster" represents the cluster (categorical)

"Strata" represents the stratification covariate of interest (categorical)

"Time" represents the measurement period of the observation (categorical)

"Outcome" represents the outcome measurement (continuous)

***Pre-processing**

generate notreat=1-Treat

egen clusweek = group(Cluster Time)

egen clustreat=group(Cluster Treat)

egen trtweek=group(Treat Time)

egen clustrtweek=group(Treat Time Cluster)

***Model estimates**

**Naive unadjusted for time model*

melogit Outcome i.Treat || Cluster:, or

estat icc

**Basic H and H model*

melogit Outcome i.Treat i.Time || Cluster:, or

estat icc

**Model extension A:* *Fixed strata by time interaction*

melogit Outcome i.Treat i.Time i.nStrata i.nStrata#i.Time || Cluster:, or

estat icc

**Model extension B:* *Random time by cluster interaction*

melogit Outcome i.Treat i.Time|| Cluster: || clusweek: , or

estat icc

*Note that clusweek represents the cluster-period of the measurement and this is nested within cluster.

**Model extension C: Fixed strata by treatment interaction*

melogit Outcome i.Treat i.Time i.nStrata i.nStrata#i.Treat || Cluster:, or

estat icc

**Model extension D:* *Random cluster by treatment interaction (melogit did not converge)*

xtmelogit Outcome i.Treat i.Time|| Cluster: trt notreat, or cov(uns)

local var_r_trt = exp(2*_b[lns1_1_1:_cons])

local var_r_control = exp(2*_b[lns1_1_2:_cons])

local corr_r_trt_control = tanh(_b[atr1_1_1_2:_cons])

local cov_r_trt_control = `corr_r_trt_control'*sqrt(`var_r_trt'*`var_r_control')

local var_error = _pi^2/3

noi di "ICC in treated = " `var_r_trt'/ (`var_r_trt' + `var_error')

noi di "ICC in control = " `var_r_control'/ (`var_r_control' + `var_error')

noi di "ICC mixed = " `cov_r_trt_control'/( sqrt(`var_r_trt' + `var_error')*sqrt(`var_r_control' + `var_error') )

**Model extension E:* *Fixed treat by time interaction*

melogit Outcome i.Treat i.Time i.Treat#i.Time || Cluster: , or

estat icc
